# Gut microbiota varies by opioid use, circulating leptin and oxytocin in African American men with diabetes and high burden of chronic disease

**DOI:** 10.1371/journal.pone.0194171

**Published:** 2018-03-29

**Authors:** Elena Barengolts, Stefan J. Green, Yuval Eisenberg, Arfana Akbar, Bharathi Reddivari, Brian T. Layden, Lara Dugas, George Chlipala

**Affiliations:** 1 Department of Medicine, University of Illinois Medical Center, Chicago, Illinois, United States of America; 2 Department of Medicine, Jesse Brown VA Medical Center, Chicago, Illinois, United States of America; 3 DNA Services Facility, Research Resources Center, University of Illinois, Chicago, Illinois, United States of America; 4 Research and Development Division, Jesse Brown VA Medical Center, Chicago, Illinois, United States of America; 5 Department of Public Health Sciences, Loyola University, Maywood, Illinois, United States of America; 6 Core for Research Informatics, Research Resources Center, University of Illinois, Chicago, Illinois, United States of America; Tokai University, JAPAN

## Abstract

**Objective:**

The gut microbiota is known to be related to type 2 diabetes (T2D), psychiatric conditions, and opioid use. In this study, we tested the hypothesis that variability in gut microbiota in T2D is associated with psycho-metabolic health.

**Methods:**

A cross-sectional study was conducted among African American men (AAM) (n = 99) that were outpatients at a Chicago VA Medical Center. The main outcome measures included fecal microbiota ecology (by 16S rRNA gene sequencing), psychiatric disorders including opioid use, and circulating leptin and oxytocin as representative hormone biomarkers for obesity and psychological pro-social behavior.

**Results:**

The study subjects had prevalent overweight/obesity (78%), T2D (50%) and co-morbid psychiatric (65%) and opioid use (45%) disorders. In the analysis of microbiota, the data showed interactions of opioids, T2D and metformin with *Bifidobacterium* and *Prevotella* genera. The differential analysis of *Bifidobacterium* stratified by opioids, T2D and metformin, showed significant interactions among these factors indicating that the effect of one factor was changed by the other (FDR-adjusted p [q] < 0.01). In addition, the pair-wise comparison showed that participants with T2D not taking metformin had a significant 6.74 log2 fold increase in *Bifidobacterium* in opioid users as compared to non-users (q = 2.2 x 10^−8^). Since metformin was not included in this pair-wise comparison, the significant ‘q’ suggested association of opioid use with *Bifidobacterium* abundance. The differences in *Bifidobacterium* abundance could possibly be explained by opioids acting as organic cation transporter 1 (OCT1) inhibitors. Analysis stratified by lower and higher leptin and oxytocin (divided by the 50^th^ percentile) in the subgroup without T2D showed lower *Dialister* in High-Leptin vs. Low-Leptin (p = 0.03). Contrary, the opposite was shown for oxytocin, higher *Dialister* in High-Oxytocin vs. Low-Oxytocin (p = 0.04).

**Conclusions:**

The study demonstrated for the first time that *Bifidobacterium* and *Prevotella* abundance was affected by interactions of T2D, metformin and opioid use. Also, in subjects without T2D *Dialister* abundance varied according to circulating leptin and oxytocin.

## Introduction

A role for the gut microbiota in human health is increasingly recognized. A healthy and diverse gut microbiota appears critical for normal growth while alteration (“dysbiosis”) can result in obesity and type 2 diabetes (T2D), or malnutrition [1–4]. Similarly, microbiota appears important for social functioning whereas dysbiosis is implicated in maladaptive behaviors [5,6]. Gut microbiota is suggested as a potential mechanistic link between many psycho-metabolic conditions including obesity, T2D, anorexia, depression, and drug addiction [1–4,7,8]. The bifidobacteria and lactobacilli, particularly, have been singled out as beneficial for psycho-metabolic health [9–11]. Although not yet indicated or approved for the management of any specific disease, bifidobacteria and lactobacilli are suggested as major producers of gamma-aminobutyric acid (GABA), an important neuromodulator [12]. The GABA analogs, including pregabalin, gabapentin, baclofen, and valproic acid are FDA-approved drugs for treatment of psychiatric, gastro-intestinal, and diabetes-related disorders [13].

Type 2 diabetes and obesity are major causes of morbidity and mortality and are reaching epidemic proportions through the world [13]. Psychiatric co-morbidities contribute to complexity of the pathogenesis and management of both T2D and obesity [13]. Similar to T2D, opioid use and addiction are reaching epidemic proportions and are evolving as important causes of morbidity and mortality. Opioid addiction co-morbidity complicates diabetes management and increases mortality [13,14]. Mortality rates of dependent opioid users are approximately 15-fold higher than age- and sex-matched controls [14]. Moreover, opioid use and addiction appear particularly pertinent to T2D and obesity as similar pathophysiologic pathways are implicated in drug and food addiction, likely involving the gut-brain axis [15]. The neuropeptide hormone oxytocin (OXT) is emerging as an important part of the gut-brain axis and as a promising treatment of obesity, T2D, and addiction [2,16–20]. OXT synthesis in the brain hypothalamus is regulated by leptin, insulin, and dopaminergic pathways, which are particularly relevant to obesity, glucose metabolism, and addiction [16,17]. In addition, OXT signaling may be connected to gut microbiota. For example, feeding mice with *Lactobacillus reuteri* resulted in a significant up-regulation of plasma levels of oxytocin and associated surge in grooming behavior [2,18].

Information on gut-brain axis linking T2D and obesity with food and drug addiction is exponentially increasing, yet multiple questions remain. Majority of the data are coming from animal studies with few data available from human cohorts or trials [15–20]. Difficulties of observing any associations are logical in real world cohorts in patients with multiple conditions and confounding factors including use of metformin in diabetes [1–3]. It is important, however, to attempt establishing associations of gut microbiota with psycho-metabolic health in complex cohorts to test the applicability of data emerging from rodent studies, for providing mechanistic insight and for planning randomized trials. The present study tested the hypothesis that gut-brain axis is involved in associations of gut microbiota with psycho-metabolic health in men with high burden of chronic conditions. Specifically, this analysis tested the hypothesis of whether bifidobacteria and lactobacilli were 1) linked to T2D and opioid use, and 2) linked to circulating leptin and oxytocin representing obesity-T2D and pro-social psychological hormone markers, respectively [16–18]. To make the data representative of real world population, patients with T2D drug-naïve and T2D treated with metformin were included since metformin had been recognized as the first line and most widely used treatment for T2D [13].

## Materials and methods

### Design and subjects

This study was a cross-sectional study of African American men (AAM) (n = 99), that received their care from an urban Veteran Health Administration Medical Center. This study was a part of the Glucose tolerance and vitamin D in African American Male veterans (GluDAAM) cohort study that evaluated glucose metabolism biomarkers in AAM. The inclusion criteria were glycohemoglobin A1c (HbA1c) < 5.7% without T2D or 6.5–7.4% with T2D, age 35–70 years, body mass index (BMI) 22–39.9 kg/m^2^, and 25OH-vitamin D (25OHD) < 30 ng/ml. Exclusion criteria were chronic kidney disease (stages 3b, 4, and 5), chronic glucocorticoid use (3 months or longer), taking non-metformin antihyperglycemics, and presence or history of significant health conditions requiring recent (within 6 months) hospitalization.

The subjects came for a single study visit where biometric and biochemical measures were done. The past medical history (PMH) and opioid use (“No” or “Yes”) was confirmed by the review of the electronic medical records as previously described [21]. Opioid use was defined as “Yes” if during review of medical record two parameters were present: 1) the participant was under care of psychiatric care professional, and 2) psychiatric care professional established the diagnosis of opioid use disorder as official diagnosis in medical record. The DSM-4 diagnostic criteria for Opioid Use Disorder were used by psychiatric care professionals: “A problematic pattern of opioid use leading to clinically significant impairment or distress.” The biometric measurements and calculations (age-adjusted Charlson index) were performed as previously [21]. The study was approved by the Jesse Brown VA Medical Center Institutional Review Board and each subject signed the informed consent. The recruitment dates were from December 01, 2013 to April 15, 2016.

### Analytical methods and glycemic indice calculations

Biochemistry and hormonal assays were performed in the clinical laboratory and the core research laboratory applying laboratory standards of care and references and indices calculated as previously described [7,21]. Metabolites and hormones included HbA1c, fasting plasma glucose (FPG), insulin, C-peptide, proinsulin, lipid panel (total cholesterol, triglycerides, HDL, LDL), hormones (testosterone, leptin, oxytocin), C-reactive protein, and 25-hydroxyvitamin D. Calculations for glycemic indices were based on oral glucose tolerance test (OGTT) under dynamic, i.e. postprandial conditions. Insulin sensitivity was assessed by Oral Glucose Insulin Sensitivity (OGIS) based on modeling provided online http://webmet.pd.cnr.it/ogis/ in ml×min-1×m-2. Insulin secretion was assessed by Insulinogenic index-30 [(insulin at 30 min—fasting insulin)/(glucose at 30 min—fasting glucose)]. In addition, OGTT was used to calculate area-under-the-curve (AUC) glucose and AUC insulin. The formulas and methods had been validated previously against the ‘gold-standard’ method of glucose clamp with and without tracers and are commonly used in research related to diabetes. Oxytocin was measured in a clean catch urine sample at the University of Wisconsin-Madison’s Wisconsin National Primate Research Center (WNPRC) with Assay Design ELISA kits (Enzo Life Sciences, Ann Arbor, MI) [22].

### Microbial community evaluation

Participants were provided with a plastic device to collect stool samples, which were stored at -80°C until extraction. Genomic DNA was extracted, processed for microbial community analysis using PCR amplification, followed by high-throughput sequencing on an Illumina MiSeq sequencer as described previously [7,23]. Briefly, the widely used primer sets 341F/806R, targeting the V3-V4 variable regions of the 16S rRNA gene of Bacteria, was used. A two-stage PCR or “targeted amplicon sequencing (TAS)” approach was performed to generate amplicon libraries. In the first stage PCRs were performed in 10 μl reaction volumes using 2X MyTaq HS Mix (Bioline, Taunton, MA). Subsequently, a second PCR reaction was established, with one μl of amplification product from the first stage used as input to the second reaction. The primers for the second stage amplifications were the AccessArray barcoding system primers (Fluidigm, South San Francisco, CA), containing Illumina sequencing adapters, sample-specific barcodes, and CS1 and CS2 linkers[7,23]. Final PCR products were purified and equalized using SequalPrep Normalization Plate Kit (Thermo Fisher Scientific), according to the manufacturer’s instructions. Samples were pooled in equimolar ratio and quantified using a Qubit 2.0 fluorometer. Sequencing was performed on an Illumina MiSeq sequencer using standard V3 chemistry with paired-end, 300 base reads. Fluidigm sequencing primers, targeting the CS1 and CS2 linker regions, were used to initiate sequencing. Library preparation and sequencing was performed at the DNA Services Facility at the University of Illinois at Chicago [7,23].

#### Basic sequence processing

Forward and reverse reads were merged using the software package PEAR [24]. Primer sequences were identified using Smith-Watermann alignment. Reads that lacked either primer sequence were discarded. Sequences were then trimmed based on quality scores using a modified Mott algorithm with PHRED quality threshold of p = 0.01. After trimming any sequences less than 275 bp were discarded. Chimeric sequences were identified using the USEARCH61 algorithm with the GreenGenes 13_8 reference sequences [7]. QIIME v1.8 was used to generate OTU tables and taxonomic summaries [7]. Briefly, the resulting sequence files were merged with sample information. Operational taxonomic unit (OTU) clusters were generated in a *de novo* manner using the UCLUST algorithm with a 97% similarity threshold. Taxonomic annotations for each OTU were determined using the UCLUST algorithm and GreenGenes 13_8 reference with a minimum similarity threshold of 90% [7]. Taxonomic and OTU abundance data were merged into a single OTU table. Prior to any analyses the OTU table was filtered to remove any sequences from mitochondria or chloroplasts and then rarified to a depth of 5600 counts per sample. The filtered and rarified OTU table was then used to generate summaries of absolute abundances of taxa for all phyla, classes, orders, families, genera, and species [7].

### Statistical analysis

#### Metabolic indicators

Statistical analysis of metabolic indicators was performed as described previously [7,21]. The groups were specified a priori. Descriptive statistics were done for the whole group and for subjects without T2D (DM-) and with (DM+). This analysis was dedicated to interactions and/or associations of microbiota with psycho-metabolic health. Therefore, the groups were stratified by use of opioids (No/Yes as Op-/Op+), T2D (DM-/DM+), and use of metformin (MF-/MF+). Six identified groups were as follows: Gr1 = Op-/DM-/MF-, Gr2 = Op-/DM+/MF-, Gr3 = Op-/DM+/MF+, Gr4 = Op+/DM-/MF-, Gr5 = Op+/DM+/MF-, Gr6 = Op+/DM+/MF+. Data were described as mean ± standard deviation (SD) for continuous variables or number (percent) for categorical variables. For categorical variables data were number (%) for “yes” answer, and the Fisher’s exact test and logistic regression, were used to denote statistical significance. For continuous variables ANOVA with Bonferroni adjustment, and linear regression were used. For the logistic and linear regressions; the reference category was Op-/DM-/MF-, and p<0.05 was considered statistically significant. All metabolic analyses were performed in STATA v.14 (College Station, TX, USA).

#### Microbiota analysis

For each microbiota sample and taxon, raw sequence counts from the rarefied dataset were used for analysis. The values for the taxa were reported as the total sequence counts. Prior to group testing and correlation analyses, all taxonomic summaries were filtered to remove any taxon with an abundance of less than 1% of the total abundance in the dataset.

Shannon and Bray-Curtis indices were calculated in R using the vegan library. The rarefied genus data, taxonomic level 6, were used to calculate both indices. For Bray-Curtis indices, the rarefied genus data were filtered to remove any taxon with an abundance of less than 1% of the total abundance in the dataset and sequence counts from the filtered data were transformed using a log_10_(*x*+1) transformation. Comparisons of similarities among groups were performed using ANOSIM. The Krukal-Wallis one-way analysis of variance was used to compare Shannon’s diversity indices. Comparisons of relative sequence abundance among groups were performed using Kruskal-Wallis one-way analysis of variance using the group_significance.py script within the QIIME v1.8 package. The differences in microbiota taxonomic relative abundance were compared using Mann-Whitney nonparametric test. For group significance testing, summaries for all taxonomic levels were used except for ambiguous taxa, i.e. “Other” or unnamed. False discovery rate (FDR)-corrected P values were estimated with the significance set at P < 0.05 for Mann-Whitney test [7]. Correlations were tested between marker levels determined via biochemical and hormonal assays and all taxonomic units using Kendall Tau (τ) test of correlation. All statistical analyses were performed using R 3.2.3 statistical software.

In addition, to assess interaction between factors that appear to significantly influence microbiota (opioid and metformin use), differential analysis was performed. Prior to differential analysis, unrarefied taxonomic summaries were filtered to remove any samples with less than 5000 total sequence counts and any taxon with an abundance of less than 1% of the total abundance in the dataset. Differential analyses of taxa as compared with factors were performed using edgeR [25]. Briefly, data were normalized using a trimmed mean of M-values (TMM). Microbiota abundance was expressed as the log count per million (logCPM) number, which was the base or average normalized abundance across all samples tested showing if that particular taxon was a relatively high or low abundance taxon. Normalized data were then fit using a negative binomial generalized linear model and statistical tests were performed using a likelihood ratio test. FDR-adjusted p values were calculated using the Benjamini-Hochberg false discovery rate (FDR) correction [26]. All FDR-adjusted p values were designated q values.

Data access. The amplicon sequence data from this study have been submitted to the NCBI Sequence Read Archive (http://www.ncbi.nlm.nih.gov/Traces/sra/sra.cgi) under the BioProject PRJNA389481.

## Results

### Bio-clinical subject characteristics

Assessment of all subjects showed high rate of smoking (35%), opioid use (45%), and overweight/obesity (78%). There was high rate of chronic (95%), including psychiatric (65%) disorders and use of medications (85%) showing high burden of chronic disease. Of 49 subjects with T2D, 30 used metformin. All subjects with T2D were overweight or obese. Comparison of six subgroups based on opioid use, diagnosis of T2D, and use of metformin showed some differences (Table 1). Particularly, indices related to obesity (body weight, BMI, WHR, and fat percent), T2D (HbA1c and glycemic indices), hormones (testosterone, leptin), and T2D-related co-morbidities (obesity, hypertension, hyperlipidemia, and any psychiatric disorders, and PTSD) were different among the groups.

**Table 1:** Subject characteristics.

Characteristics	Op-/DM-	Op-/DM+/MF-	Op-/DM+/MF+	Op+/DM-	Op+/DM+/MF-	Op+/DM+/MF+	Overall p
	N = 24	N = 11	N = 19	N = 28	N = 5	N = 6	
**General**							
Age, yr	54.6 ± 7.3	57.5 ± 6.6	58.2 ± 4.5	56.1 ± 4.3	58.4 ± 3.7	56.6 ± 4.7	0.473
SBP, mmHg	130.8 ± 13.8	136.1 ± 23.4	134.5 ± 15.8	127.8 ± 15.7	134.9 ± 8.1	142.2 ± 12.6	0.243
DBP, mmHg	76.1 ± 10.6	77.1 ± 10.1	75.7 ± 838	79.3 ± 11.9	80.1 ± 7.6	77.9 ± 10.3	0.794
Body weight, kg	83.1 ± 14.1	107.3 ± 9.7**	111.4 ± 15.4	81.3 ± 13.7	112.8 ± 14.6**	102.6 ± 15.7*	<0.0001
BMI, kg/m2	26.3 ± 4.0	35.6 ± 2.4**	35.3 ± 3.4**	27.1 ± 3.8	36.2 ± 2.9**	34.6 ± 2.9**	<0.0001
WHR	0.92 ± 0.06	1.01 ± 0.04**	1.05 ± 0.08**	0.94 ± 0.06	1.03 ± 0.04**	1.04 ± 0.09**	<0.0001
Total Fat, %	24.8 ± 7.8	35.6 ± 4.6**	36.1 ± 5.7**	25.3 ± 7.6	35.6 ± 3.4**	32.8 ± 5.8**	<0.0001
Android Fat, %	30.4 ± 12.3	47.3 ± 6.2**	47.6 ± 7.9**	30.5 ± 11.9	47.2 ± 4.8**	43.5 ± 7.5**	<0.0001
Gynoid Fat, %	26.1 ± 7.1	34.5 ± 4.6**	35.4 ± 5.5**	26.5 ± 7.4	34.7 ± 4.3*	32.5 ± 5.5**	<0.0001
A/G fat ratio	1.1 ± 0.2	1.4 ± 0.1**	1.3 ± 0.1**	1.1 ± 0.2	1.4 ± 0.2**	1.3 ± 0.1*	0.001
Charlson index	1.5 ± 1.2	1.9 ± 1.4	2.2 ± 1.3	2.0 ± 1.6	2.7 ± 2.6	2.4 ± 1.7	0.39
N of all meds	7.7 ± 6.8	7.5 ± 4.3	10.3 ± 6.6	9.5 ± 8.4	9.8 ± 5.1	9.1 ± 7.0	0.808
**Glycemic**							
HbA1c, %	5.2 ± 0.3	6.7 ± 0.3**	6.9 ± 0.4**	5.3 ± 0.6	6.7 ± 0.3**	6.7 ± 0.2**	<0.0001
FPG, mg/dL	94.0 ± 16.7	112.5 ± 14.7**	126.2 ± 20.0**	94.8 ± 11.1	137.7 ± 34.4**	115.6 ± 17.1**	<0.0001
F Insulin, mIU/L	10.7 ± 8.0	28.0 ± 14.0*	20.1 ± 10.4	9.8 ± 10.4	61.2 ± 81.1**	19.5 ± 9.4	0.0001
F C-peptide, pmol/L	385.1 ± 377.2	1209.2 ± 575.0**	998.3 ± 355.9**	400.3 ± 393.1	1549.5 ± 731.3**	752.4 ± 244.3*	<0.0001
F proinsulin, pmol/L	16.6 ± 5.8	31.8 ± 11.6	37.8 ± 16.4**	21.9 ± 24.9	53.8 ± 35.3**	28.9 ± 13.8	0.0005
OGIS	451.1 ± 73.1	343.1 ± 81.5**	330.8 ± 65.8**	438.2 ± 86.8	279.7 ± 54.4**	298.6 ± 114.5**	<0.0001
Insulinogenic index	1.0 ± 0.7	1.1 ± 1.0	0.5 ± 0.4	1.5 ± 1.5	2.0 ± 2.0*	0.7 ± 0.4	0.012
AUC glucose x1000	21.7 ± 6.1	28.7 ± 5.7**	34.0 ± 8.1**	20.0 ± 4.5	38.6 ± 12.1**	32.3 ± 10.9**	<0.0001
AUC insulin x1000	9.1 ± 5.6	15.6 ± 7.6*	11.0 ± 4.8	9.3 ± 7.9	25.8 ± 18.6**	12.4 ± 5.5	<0.0001
**Lipids, chemistry**							
TC, mg/dL	179.4 ± 34.9	161.7 ± 45.3	159.9 ± 45.3	166.1 ± 34.7	167.6 ± 39.6	150.3 ± 49.6	0.534
Triglyceride, mg/dL	110.4 ± 89.6	120.6 ± 61.5	171.2 ± 105.0*	94.8 ± 49.8	193.1 ± 118.6*	109.6 ± 25.5	0.011
HDL, mg/dL	62.8 ± 19.4	46.3 ± 9.9**	44.0 ± 9.9**	56.6 ± 19.0	42.9 ± 8.3**	44.9 ± 11.0**	0.0006
LDL, mg/dL	96.4 ± 25.8	91.5 ± 37.1	90.6 ± 28.0	90.6 ± 34.2	95.3 ± 35.7	83.7 ± 41.6	0.948
Creatinine, mg/dL	1.0 ± 0.2	1.1 ± 0.2	1.1 ± 0.2	1.0 ± 0.1	1.2 ± 0.2	1.0 ± 0.2	0.093
AST/ALT ratio	0.9 ± 0.3	0.7 ± 0.4	0.7 ± 0.5	0.9 ± 0.4	0.7 ± 0.5	0.7 ± 0.2	0.38
**Hormones**							
Testosterone, ng/dl	404.6 ± 171.5	262.2 ± 115.7**	229.5 ± 120.2**	344.1 ± 125.4	266.8 ± 90.9*	293.4 ± 165.1*	0.002
Leptin, ng/ml	10.5 ± 11.9	27.6 ± 14.8**	30.4 ± 20.0**	10.7 ± 8.9	37.1 ± 13.2**	26.3 ± 18.6**	<0.0001
Oxytocin, pg/mg	8.1 ± 7.2	5.7 ± 5.3	6.4 ± 7.9	10.0 ± 7.0	5.8 ± 5.1	9.3 ± 4.7	0.219
25OHD, ng/mL	17.1 ± 6.1	12.1 ± 7.4	17.1 ± 7.8	15.4 ± 6.3	12.4 ± 6.9	18.2 ± 7.1	0.167
CRP, mg/L	6.0 ± 10.0	9.9 ± 13.0	4.6 ± 2.9	4.9 ± 5.6	3.9 ± 4.2	3.8 ± 5.0	0.415
**Medical, N [%]**							
Current smoking	4 [16.7]	4 [36.4]	8 [44.4]	15 [53.5]*	2 [25]	2 [22.2]	0.114
Overweight	9 [33.3]	0 [0.0]	1 [5.3]*	13 [46.4]	0 [0.0]	0 [0.0]	<0.001
Obesity	4 [16.7]	11 [100.0]	18 [94.7]**	5 [17.9]	8 [100.0]	9 [100.0]	<0.001
Hypertension	11 [50.0]	8 [72.7]	18 [94.7]**	13 [54.2]	5 [71.4]	7 [77.8]	0.02
Hyperlipidemia	9 [37.5]	6 [54.6]	15 [79.0]**	11 [39.9]	6 [75.0]	8 [89.0]*	0.007
CVD	3 [12.5]	1 [9.1]	5 [26.3]	4 [14.3]	3 [37.5]	2 [22.2]	0.495
OSA	6 [25.0]	3 [27.3]	11 [57.9]	6 [35.3]	3 [37.5]	3 [33.3]	0.168
Any psych disorder	15 [62.5]	4 [36.4]	10 [52.6]	26 [92.9]*	4 [50.0]	6 [66.7]	0.003
Depression	11 [45.8]	3 [27.7]	3 [15.8]*	13 [46.4]	0 [0.0]	4 [44.4]	0.041
PTSD	8 [33.3]	1 [9.1]	0 [0.0]	13 [46.4]	2 [25.0]	1 [11.1]	0.002
Other psych	7 [29.2]	2 [18.2]	4 [21.1]	9 [32.1]	0 [0.0]	2 [22.2]	0.565
Any medication	18 [75.0]	11 [100.0]	18 [94.7]	24 [85.7]	8 [8.1	9 [100.0]	0.192

Data are Mean ± SD or N [%]

*p<0.05, and

**p<0.01.

For categorical variables data are number [%] for “yes” answer, Fisher’s exact test was used for composite overall p (p-value) and logistic regression was used for between group comparisons with Op-/DM- serving as reference group. For continuous variables ANOVA with Bonferroni adjustment was used for a composite overall p-value, and linear regression was used for between group comparisons with Op-/DM- serving as reference group. Abbreviations: 25OHD = 25-hydroxyvitamin D, HbA1c = hemoglobin A1c, A/G = Android/Gynoid, AUC = area under the curve, BMI = Body mass index, Charlson index = index of chronic disease, Cr = Creatinine, CRP = C reactive protein, CVD = Cardiovascular disease, DBP = Diastolic blood pressure, DM = type 2 diabetes mellitus, F = fasting, FPG = fasting plasma glucose, N = number, OGIS = Oral glucose insulin sensitivity (Mari’s index), Op = Opioid use, OSA = Obstructive sleep apnea, Psych = psychiatric, PTSD = Post-traumatic stress disorder, SBP = Systolic blood pressure, TC = Total cholesterol, WHR = waist to hip ratio.

### Sequencing coverage and estimation of fecal bacterial diversity

**Fig 1:**
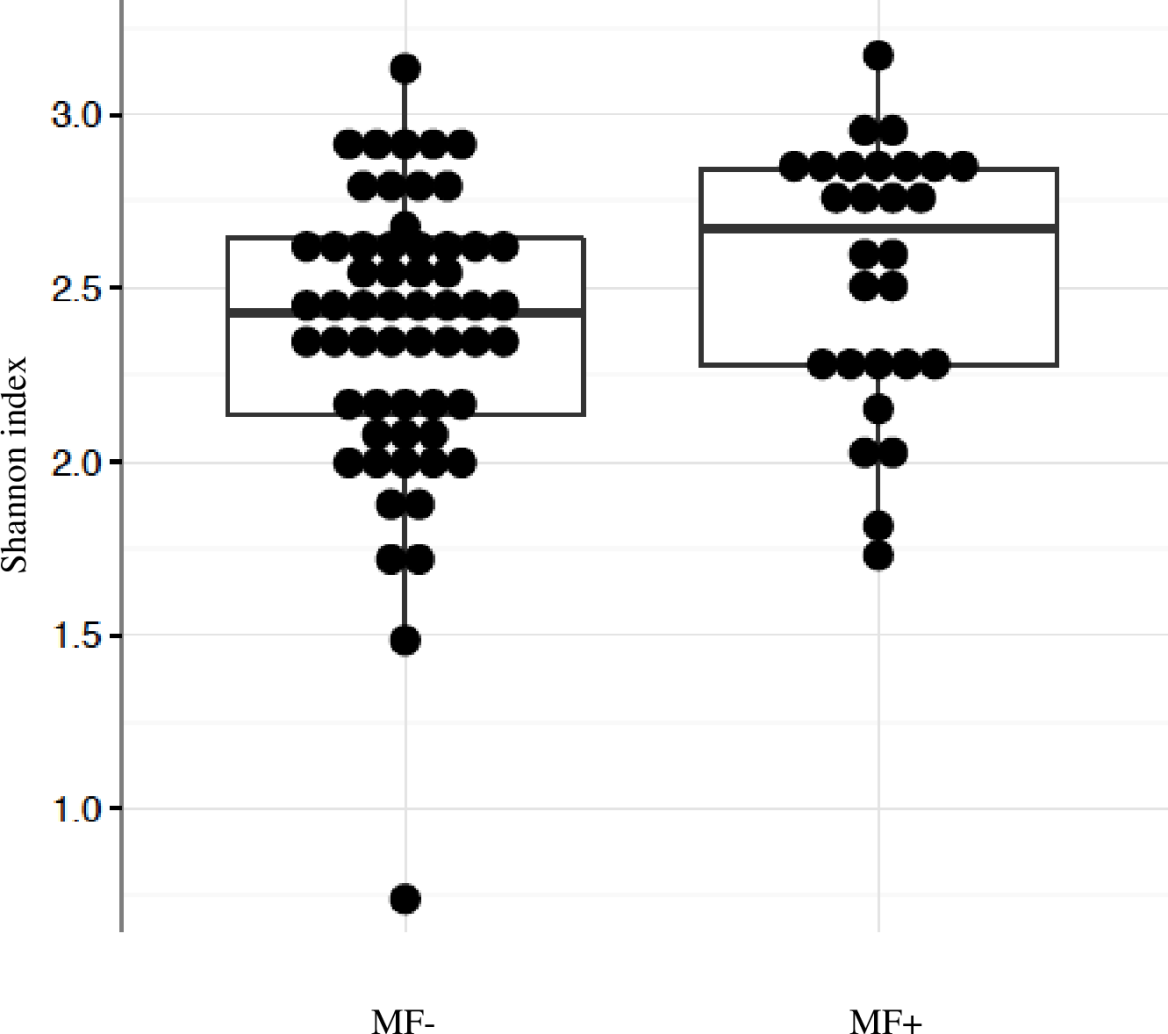
Shannon index of alpha diversity. Pairwise Mann-Whitney test was used to compare alpha diversity estimates, MF- vs. MF+ (p = 0.05). MF- group includes DM- plus DM+/MF- (n = 69), MF+ group includes DM+/MF+ (n = 30). Abbreviations: DM = type 2 diabetes mellitus, MF = Metformin.

**Fig 2:**
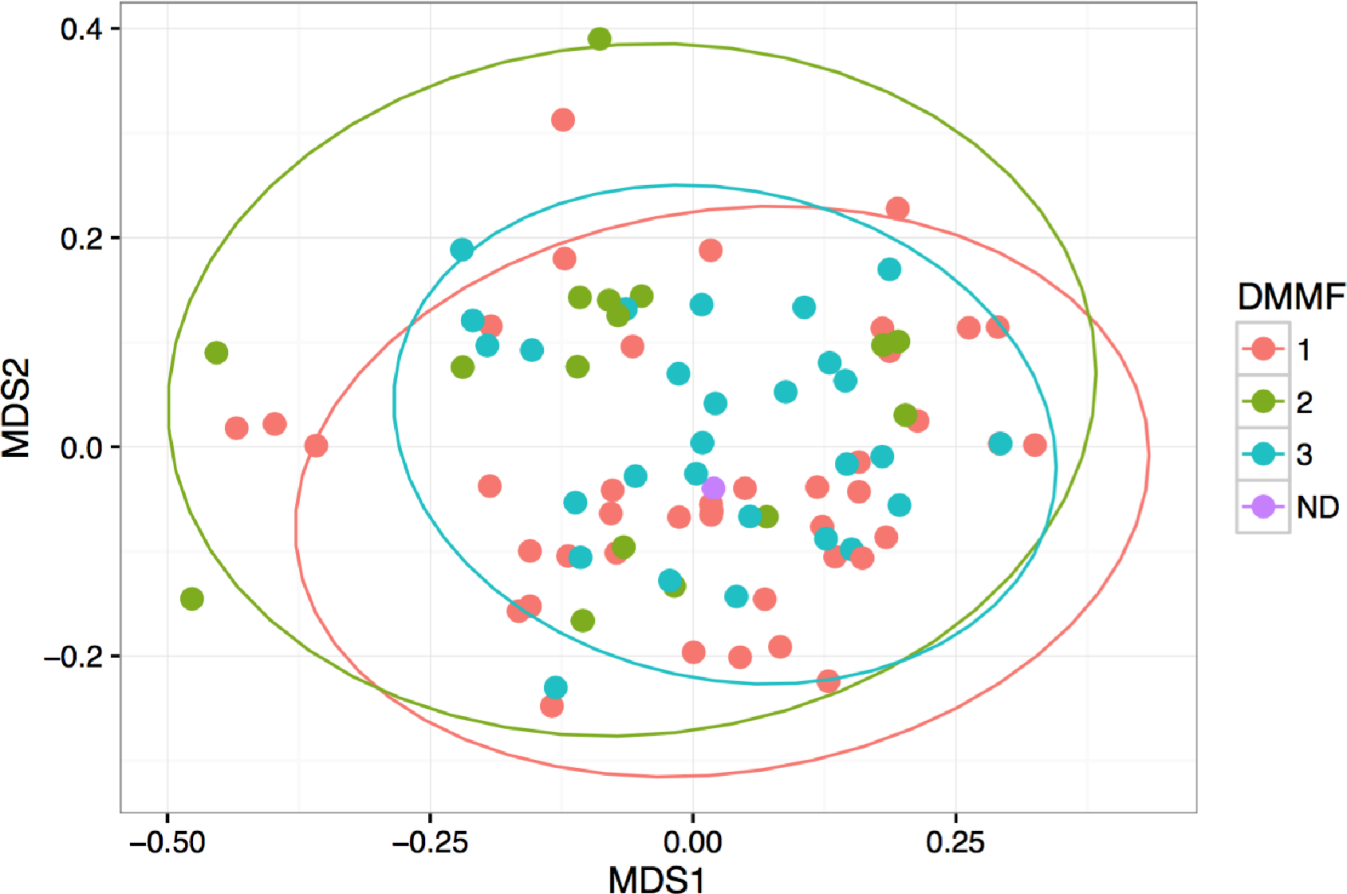
Non-metric multidimensional scaling (NMDS) ordination plot. The NMDS is based on Bray-Curtis dissimilarities between OTU-level microbiota communities in three groups: 1 = DM-, 2 = DM+/MF-, 3 = DM+/MF+, ND = not defined. Pairwise ANOSIM comparison showed a trend for DM+/MF- vs. DM+/MF+ (p = 0.067). Abbreviations: DM = type 2 diabetes mellitus, MF = Metformin.

In this study, the bacterial composition of the fecal samples was examined using an Illumina high-throughput sequencing technique. We generated a dataset consisting of 2,639,754 total sequence read counts, and the average number of sequences obtained was 27,497. A diversity analysis based on Shannon index revealed that there was a trend for the higher diversity in the fecal samples of DM+/MF+ subjects compared with all other subjects (p = 0.05) (Fig 1). There were no differences in Shannon index between DM- vs. DM+ (2.32 vs. 2.53, p = 0.19) or within T2D subgroup between those not taking and taking metformin (2.47 vs. 2.68, p = 0.07). To compare the composition of the microbiota based on diabetes and metformin use, non-metric multi-dimensional scaling (NMDS) of a Bray-Curtis distance matrix based on the abundance of genera was employed using ANOSIM analysis. The NMDS showed a trend but did not reach statistical significance for comparison of DM- vs. DM+ groups (p = 0.09) as well as DM+/MF- vs. DM+/MF+ (p = 0.07) (Fig 2) suggesting that both groups were similar in their bacterial ecology. There were no differences for beta (between-samples) diversity for any group comparisons.

### Taxonomic composition of fecal bacterial communities

In the entire group, the four dominant phyla were Bacteroidetes (47.6%), Firmicutes (39.6%), Proteobacteria (7.7%), and Actinobacteria (2.2%). At the family level, across groups, the predominant taxa were Bacteroidaceae (30.7), Ruminococcaceae (16.2), and Lachnospiraceae (7.0%). At the genera level, 75.2% were able to be classified, of them 14 genera had abundance 1% or higher (Table 2). The most common genus when considering all groups, groups with T2D only, or groups without T2D, was *Bacteroides* then *Prevotella*. The same genera were the most common in T2D and in the entire group.

**Table 2:** Abundance of the most prevalent genera in the entire group.

Bacterial Taxon	%
p_Bacteroidetes;c_Bacteroidia;o_Bacteroidales;	
- f_Bacteroidaceae;g_Bacteroides	30.7
- f_Prevotellaceae;g_Prevotella	6.7
- f_Porphyromonadaceae;g_Parabacteroides	3.4
- f_Paraprevotellaceae;g_Prevotella	1.2
p_Firmicutes;c_Clostridia;o_Clostridiales;	
- f_Ruminococcaceae;g_Faecalibacterium	5.9
- f_Veillonellaceae;g_Dialister	1.6
- g_Phascolarctobacterium	1.4
- f_Ruminococcaceae;g_Ruminococcus	1.4
- -g_Oscillospira	1.3
- f_Lachnospiraceae;g_Lachnospira	1.1
- c_Erysipelotrichi;o_Erysipelotrichales;f_Erysipelotrichaceae;g_Catenibacterium	2.3
p_Proteobacteria;c_Deltaproteobacteria;o_Desulfovibrionales;f_Desulfovibrionaceae;g_Desulfovibrio	1
- c_Betaproteobacteria;o_Burkholderiales;f_Alcaligenaceae;g_Sutterella	3.2
p_Actinobacteria;c_Actinobacteria;o_Bifidobacteriales;f_Bifidobacteriaceae;g_Bifidobacterium	1.4

Data are percent (%) relative abundance of taxa in the entire group. Abbreviations: p = phyla, c = class, o = order, f = family, g = genus.

### Comparison of the gut microbiota between groups without and with diabetes

Gut microbial abundance varied depending on the existence of T2D and metformin use. In subjects with DM+ vs. DM-, increased abundance was observed at the genera level for *Dialister* and *Lachnospira*, both from phylum Firmicutes, with taxon relative sequence abundance DM+ vs. DM- 73 vs. 105 (p<0.01) for *Dialister* and 61 vs. 64 (p<0.05) for *Lachnospira*, respectively. Since it was expected that metformin use could change gut microbiota, T2D group was compared by use of metformin. At the genera level, specific trends for differences were observed between DM+/MF- vs. DM+/MF+ for *Catenibacterium* (phylum Firmicutes) and *Parabacteroides* (phylum Bacteroidetes) with taxon relative sequence abundance DM+/MF- vs. DM+/MF+ 52 vs. 136 for *Catenibacterium* and 128 vs. 197 for *Parabacteroides* (p<0.05 for both), respectively. Changes in specific genera associated with T2D, without metformin (e.g., *Dialister* and *Lachnospira*), were clearly distinct from those of metformin use (e.g., *Catenibacterium*, and *Parabacteroides*).

### Comparison of the gut microbiota between subgroups by opioid use

In the entire group, the opioid use subgroup was the largest among psychiatric disorders (n = 45). Grouping of subjects by opioids, T2D, and metformin showed some specific differences in microbiota abundance (Table 3). The only difference that remained significant in FDR-adjusted analysis was for genus *Bifidobacterium* (q = 0.013). There were also trends for differences in the order Lactobacillales (p = 0.02) and species *Prevotella copri* (p = 0.03).

**Table 3:** Comparison of the gut microbiota among groups.

Groups	Op-	Op-	Op-	Op+	Op+	Op+	P overall
	DM-	DM+/MF-	DM+/MF+	DM-/	DM+/MF-	DM+/MF+	
N per group	23	11	21	27	8	9	
**Class**							
Actinobacteria	110.5	3.6	28.7	82.5	285.3	47.6	<0.0001*
Order							
Bifidobacteriales	107	2.2	25.9	79.3	282	41.6	<0.0001*
Lactobacillales	41.5	4.1	57	70.6	262.9	100.5	0.02
**Family**							
Bifidobacteriaceae	107	2.2	25.9	9.3	282	41.6	<0.0001*
Paraprevotellaceae	52.4	176.3	106.5	109.1	14.4	194	0.07
**Genus**							
Prevotella; Prevotella	308.8	512.4	213.6	467.5	236	573.5	0.09
Paraprevo; Paraprevotella	25.7	137.7	71.1	79.1	0.4	136.5	0.05
Bifido; Bifidobacterium	107	2.2	25.8	79.2	281.7	41.4	<0.0001*
Veillo; Dialister	36.4	67.8	99.5	107	162	96.6	0.07
Veillo; Phascolarctobacterium	70	48.4	72	100.9	12	150.3	0.08
**Species**							
Prevotella;s_copri	242.9	349.9	143.9	349.7	162.7	433.8	0.03

Data are taxon sequence abundance adjusted relative to 5600 counts per sample. Six groups are defined by opioid use, T2D, and metformin use. The ‘P overall’ provides composite effect among six groups.

*P value for FDR = 0.013.

For statistics Mann-Whitney nonparametric test was applied and false discovery rate (FDR)-corrected P values were calculated. To assess which groups were impacted when p was significant the differential analysis was performed using edgeR. Abbreviations: Op = Opioid, DM = type 2 diabetes mellitus, MF = Metformin.

**Fig 3:**
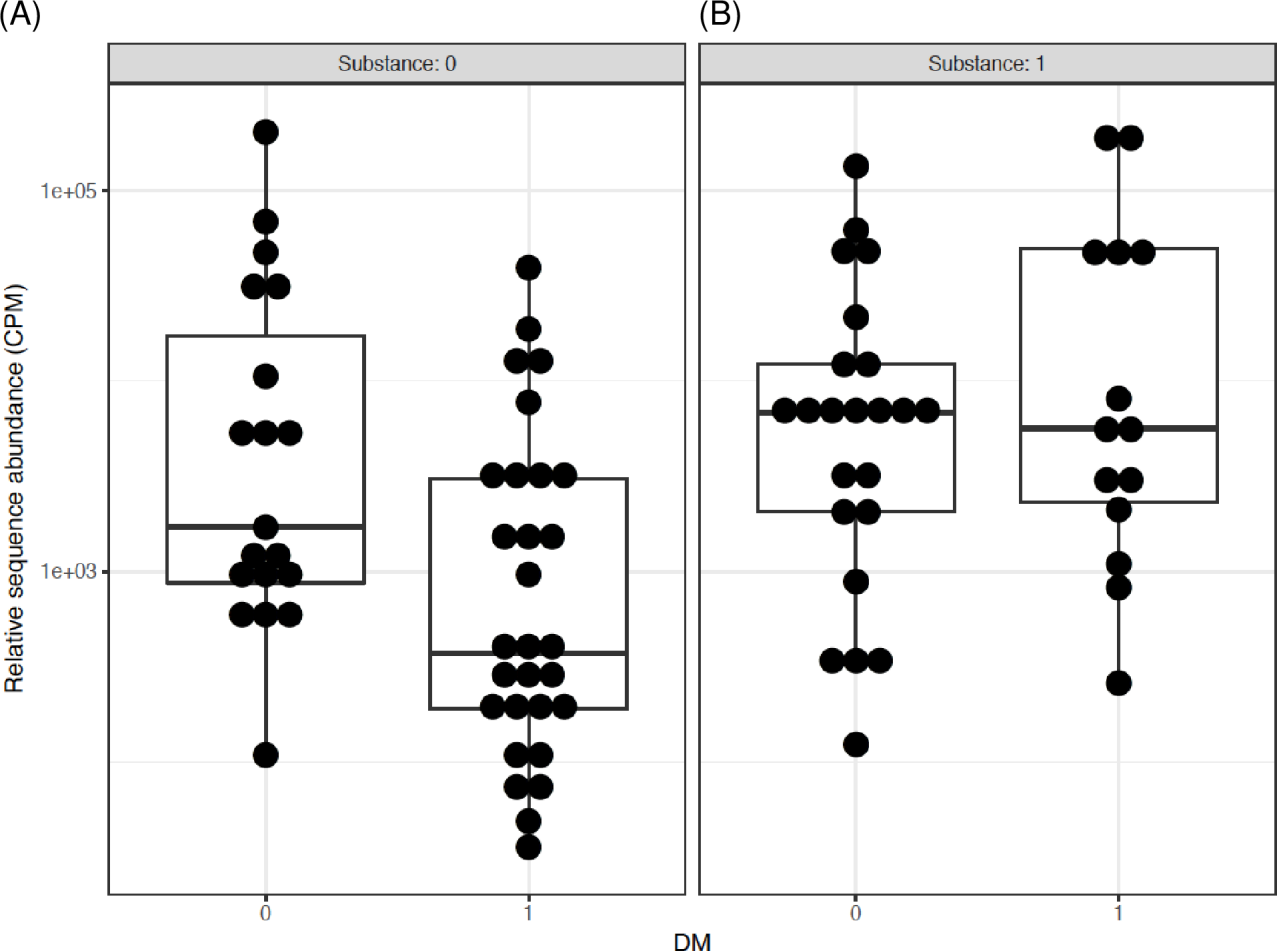
The interactive influence of T2D on Bifidobacterium genus in men using or not using opioids. Data are pair-wise comparisons for the relative sequence abundance of *Bifidobacterium*. The differential subgroup analysis was done using edgeR, the false discovery rate (FDR) adjusted p values (q values) were calculated using the Benjamini-Hochberg FDR correction. Abbreviations: 0/1 = factor absent/present. CPM = count per million, DM = type 2 diabetes, Substance = opioids. (A) 2.3 log2 fold decrease in subjects with vs. without T2D when both groups are not using opioids (q = 0.03). (B) No difference between without vs. with T2D when both groups are using opioids.

**Fig 4:**
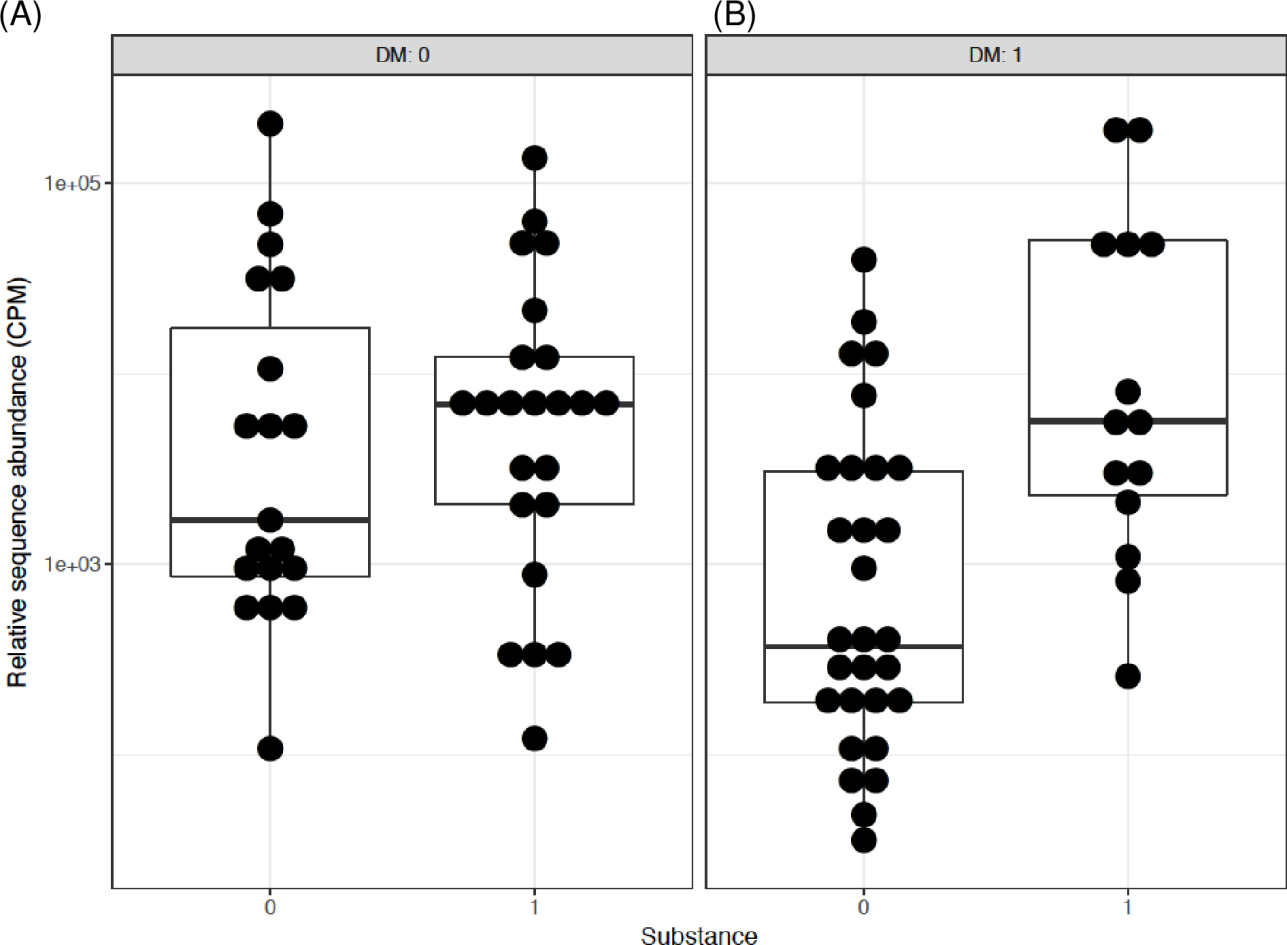
The interactive influence of opioids on Bifidobacterium genus in men with and without T2D. Data and analysis are the same as in Fig 3. (A) No difference between without vs. with opioids when both groups are without T2D. (B) 3.2 log2 fold increase in those with vs. without opioids when both groups have T2D (q = 2.5x10^-4^).

The interactions and/or associations of T2D, metformin and opioids were further assessed by differential subgroup analysis. First, the data were compared by T2D and opioid status separately. T2D but not opioid use was associated with significantly lower *Bifidobacterium* abundance, for T2D p = 2.9 x 10^−4^, q = 5.5 x 10^−3^, for opioid use p = 0.65, q = 0.88. Combination of T2D and opioids was also associated with significant difference. Both, p and q values were significant (p = 4.8 x 10^−4^, q = 9.1 x 10^−3^), indicating that the difference between the groups could not be explained by one factor alone and the effect of one factor was changed by the other. Thus, comparison was performed for all possible combinations of two factors, i.e. T2D and opioids (Figs 3 and 4). The abundance of *Bifidobacterium* was lower in T2D individuals who were not using opioids (Fig 3A). However, this effect of diabetes was not observed in the presence of opioids (Fig 3B). Similarly, the pair-wise comparison of *Bifidobacterium* showed that in participants without T2D, there was no difference in the subgroup without vs. with opioid use (Fig 4A), yet in subjects with T2D, there was 3.2 log2 fold increase in *Bifidobacterium* in those with vs. without opioids (p = 1.3 x 10^−5^, q = 2.5 x 10^−4^) (Fig 4B). There were no significant interactions for other genera.

The differential analysis of *Bifidobacterium* genus relative sequence abundance also revealed a significant interaction and association of opioid and metformin in diabetic individuals (Table 4). The comparison of each factor separately showed that each significantly affected *Bifidobacterium* abundance, for metformin p = 9.1 x 10^−4^, q = 0.02, for opioid use p = 4.6 x 10^−8^, q = 8.7 x 10^−7^. Combination of metformin and opioids also was associated with significant difference (p = 5.5 x 10^−6^, q = 1.1 x 10^−4^) suggesting a significant interaction of opioid and metformin in diabetic individuals.

**Table 4:** The interactive influence of metformin and opioids on gut microbiota in the subgroup with diabetes.

	LogFC	LogCPM	p value	q value
*Bifidobacterium*				
Interaction of Op and MF			5.5x10^-6^	1.1x10^-4^
Op-/MF+ vs Op-/MF-	3.17	11.95	3.4x10^-3^	0.03
Op+/MF+ vs Op+/MF-	-3.67	14.82	1.2x10^-3^	0.01
Op+/MF- vs Op-/MF-	6.74	14.59	1.2x10^-9^	2.2x10^-8^
Op+/MF+ vs Op-/MF+	0.53	12.40	0.51	0.88
*Prevotella*				
Interaction of Op and MF			4.3x10^-3^	0.04
Op-/MF+ vs Op-/MF-	-1.03	14.04	0.51	0.88
Op+/MF+ vs Op+/MF-	10.43	14.07	8.4x10^-4^	0.01
Op+/MF- vs Op-/MF-	-9.94	13.64	4.4x10^-3^	0.04
Op+/MF+ vs Op-/MF+	0.31	14.56	0.73	0.90
Species				
*Prevotella* unidentified				
Interaction of Op and MF			8.9x10^-3^	0.16
Op-/MF+ vs Op-/MF-	-0.65	14.42	0.63	0.92
Op+/MF+ vs Op+/MF-	10.31	14.27	1.5x10^-3^	0.03
Op+/MF- vs Op-/MF-	-9.85	13.85	0.01	0.09
Op+/MF+ vs Op-/MF+	1.13	14.62	0.46	0.85
*Bacteroides* caccae				
Interaction of Op and MF			0.03	0.19
Op-/MF+ vs Op-/MF-	-3.69	16.22	6.12x10^-5^	1.4x10^-3^
Op+/MF+ vs Op+/MF-	-0.09	13.22	0.92	0.99
Op+/MF- vs Op-/MF-	-6.09	18.24	2.8x10^-3^	0.06
Op+/MF+ vs Op-/MF+	-0.83	13.86	0.39	0.85

Data are taxon relative sequence abundance. The differential subgroup analysis was done using edgeR, the false discovery rate (FDR) adjusted p values (q values) were calculated using the Benjamini-Hochberg FDR correction. Abbreviations: CPM = count per million, FC = fold change, MF = metformin use, Op = opioid use, q = FDR-adjusted p value.

**Fig 5:**
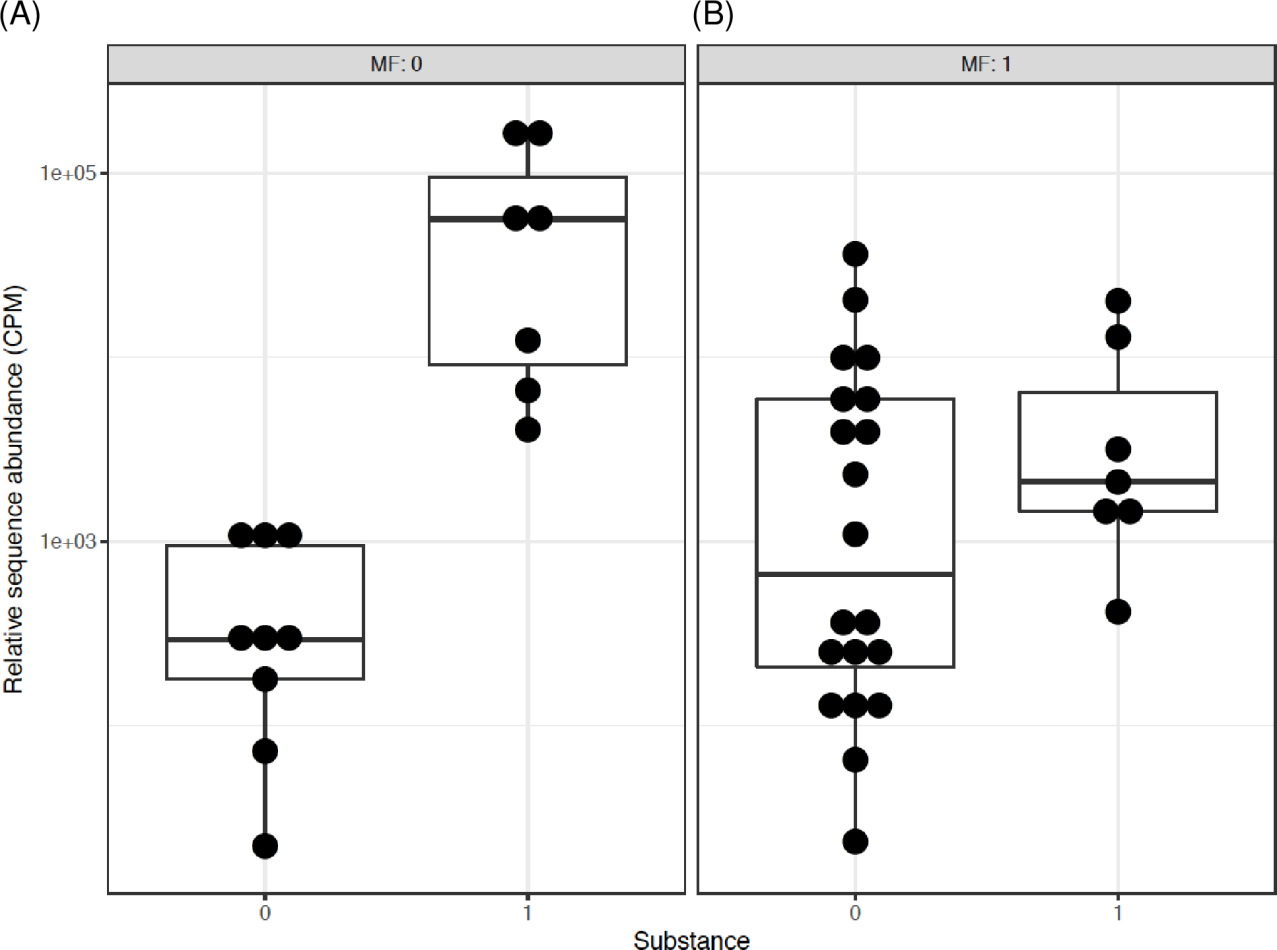
The interactive influence of metformin on Bifidobacterium genus in men with T2D and using or not using opioids. Data are pair-wise comparisons for the relative sequence abundance of *Bifidobacterium*. The differential subgroup analysis was done using edgeR, the false discovery rate (FDR) adjusted p values (q values) were calculated using the Benjamini-Hochberg FDR correction. Abbreviations: 0/1 = factor absent/present. CPM = count per million, MF = metformin, Substance = opioids. (A) 6.74 log2 fold increase in *Bifidobacterium* in opioid users vs. non-users when both groups are not taking metformin (q = 2.2 x 10^−8^). (B) No difference between opioid users vs. non-users when both groups are taking metformin.

**Fig 6:**
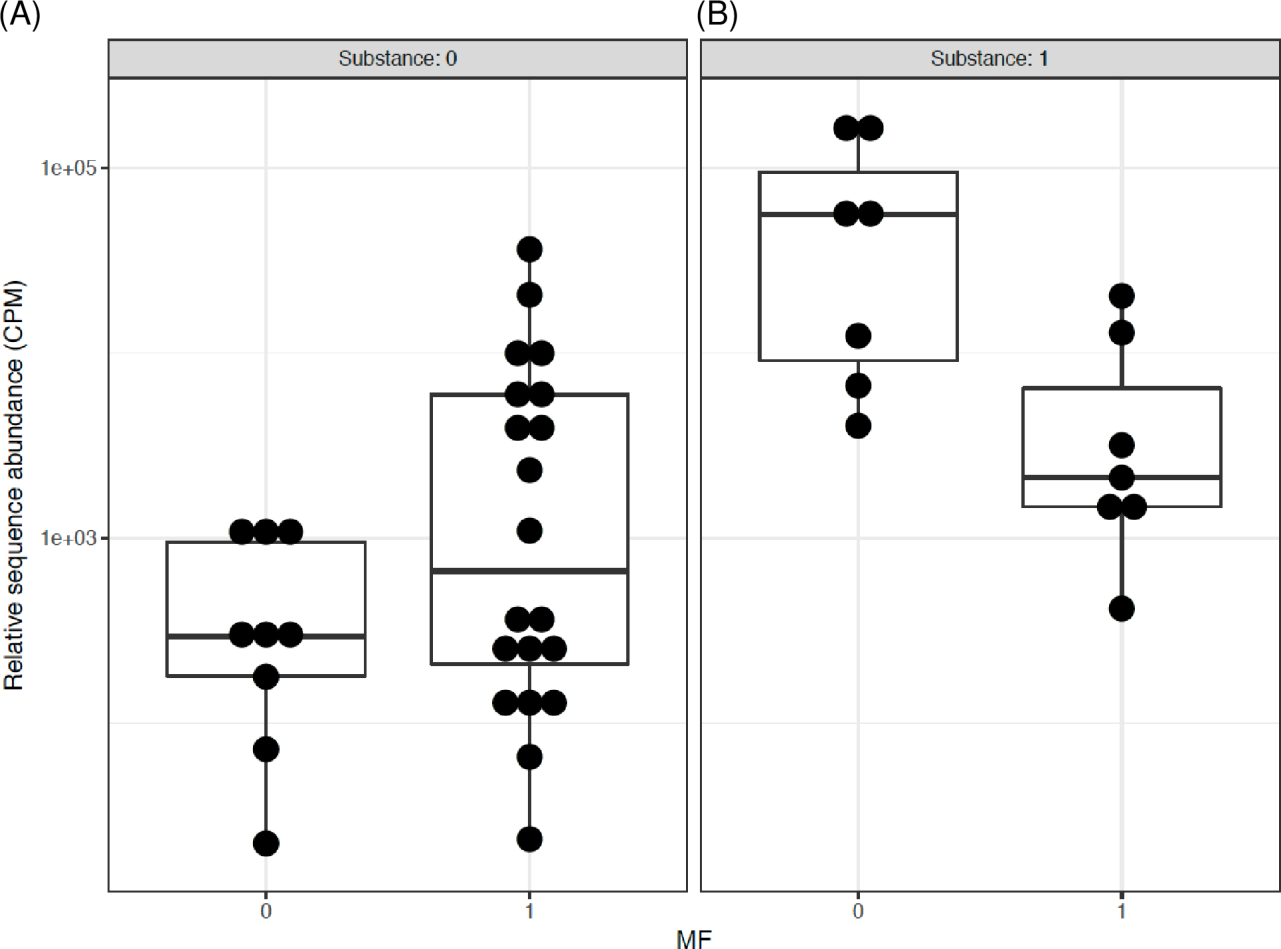
The interactive influence of opioids on Bifidobacterium genus in men with T2D and taking or not taking metformin. Data and analysis are the same as in Fig 5. (A) 3.17 log2 fold increase in *Bifidobacterium* in men taking vs. not taking metformin when both groups are not opioid users (q = 0.03). (B) 3.67 log2 fold decrease in *Bifidobacterium* in men taking vs. not taking metformin when both groups are opioid users (q = 0.01).

Further, the pair-wise comparison showed that for individuals not taking metformin there was a significant 6.74 log2 fold increase in *Bifidobacterium* abundance in opioid users as compared to non-users (p = 1.2 x 10^−9^, q = 2.2 x 10^−8^) (Fig 5A). Since metformin was not included in this pair-wise comparison, the significant “q” suggests association of opioid use with *Bifidobacterium* abundance. Contrary, there was no significant difference in *Bifidobacterium* when comparing opioid users with non-users in individuals taking metformin (log2FC = 0.53, p = 0.51, q = 0.88) (Fig 5B). Comparably, in the participants not using opioids, metformin was associated with a significant 3.17 log2 fold increase in *Bifidobacterium* relative to those not using metformin (p = 3.4 x 10^−3^, q = 0.03) (Fig 6A). The opposite relationship was observed in the participants using opioids; metformin was associated with a significant 3.67 log2 fold decrease in *Bifidobacterium* relative to those not using metformin (p = 1.2 x 10^−3^, q = 0.01) (Fig 6B), again suggesting metformin-opioid interaction.

The differential analysis of *Prevotella* genus (phylum Bacteroidetes) revealed a significant interaction of opioid and metformin in subjects with T2D (Table 4). Similar to *Bifidobacterium*, *Prevotella* abundance was associated with opioid use. The pair-wise comparison showed that for individuals not taking metformin there was a significant 9.94 log2 fold decrease in *Prevotella* in opioid users as compared to non-users (p = 4.4 x 10^−3^, q = 0.04) (Table 4). Since metformin was not included in this pair-wise comparison, the significant “q” suggests association of opioid use with *Prevotella* abundance. At the species level a trend was observed for interaction of opioids and metformin for *Prevotella* unidentified species and for *Bacteroides* caccae (Table 4).

### Comparison of the gut microbiota between groups by circulating leptin and oxytocin

**Fig 7:**
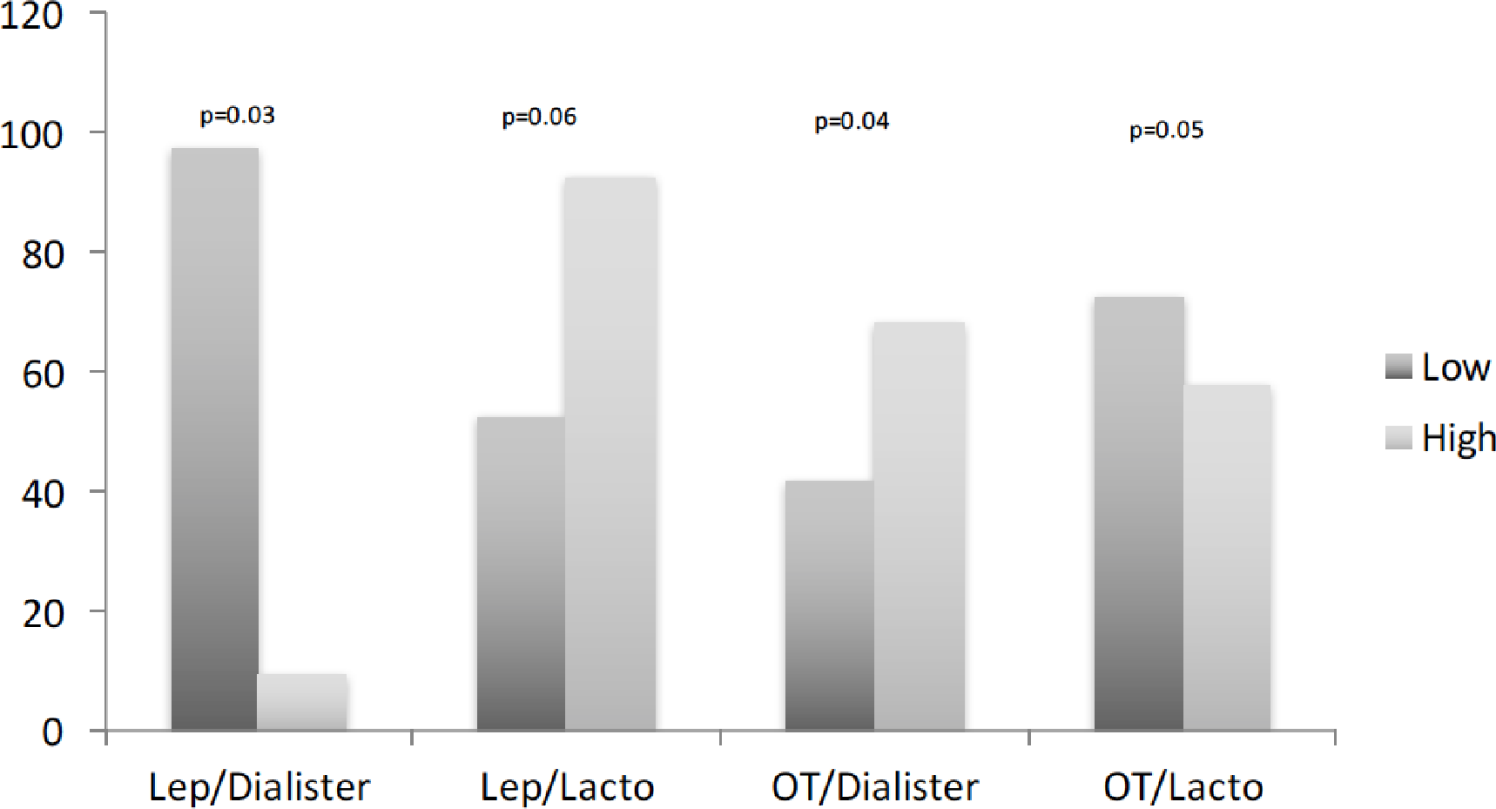
Taxa abundance based on circulating leptin and oxytocin. Data are relative counts for taxa abundance of genus *Dialister* and order Lactobacillales (Lacto) in subjects without diabetes. The subjects were divided based on low (Low) or high (High) level of Leptin (Lep) and oxytocin (OT). Pairwise Mann-Whitney test was used to compare the groups. There was lower abundance of *Dialister* in High-Leptin vs. Low-Leptin (p = 0.03), but higher abundance of *Dialister* in High-Oxytocin vs. Low-Oxytocin (p = 0.04). The opposite trends were observed for the order Lactobacillales, a higher abundance in High-Leptin vs. Low-Leptin (p = 0.06) and vice versa for oxytocin (p = 0.05).

We next explored circulating leptin and oxytocin, and their relationship to the gut microbiota, due to their essential role in obesity and behavioral aspects related to this cohort. Leptin resistance was suggested as mechanistic explanation of increased circulating leptin in obesity and T2D compared to non-obese/non-diabetic individuals, and similar variability was seen in this study. Conversely, circulating oxytocin was not different among the groups (Table 1). Comparison between lower and higher circulating leptin and oxytocin (divided by the 50^th^ percentile) in the subgroup without T2D showed some variability trends in microbiota abundance. There was lower abundance of *Dialister* in High-Leptin compared to Low-Leptin subgroup (p = 0.03). Contrary, there was higher abundance of *Dialister* in High-Oxytocin compared to Low-Oxytocin subgroup (p = 0.04). The opposite trends were observed for the order Lactobacillales, a higher abundance in High-Leptin compared to Low-Leptin subgroup (p = 0.06) and vice versa for oxytocin (p = 0.05) (Fig 7). No differences were seen in subjects with diabetes.

### Associations between bacterial populations and key circulating biomarkers

Correlation analysis was performed for the entire group and a subgroup not using metformin (n = 69) and revealed several albeit weak associations. In the entire group positive associations were found between abundance of *Dialister* and HbA1c (τ = 0.195, p = 0.013), *Ruminococcus* and fasting glucose (τ = 0.183, p = 0.014), *Catenibacterium* and OGIS (τ = 0.184, p = 0.017). In the subgroup not using metformin similar associations were shown and in addition, there was a negative correlation of *Catenibacterium* with leptin (τ = -0.254, p = 0.008) and a positive correlation of class Gammaproteobacteria with oxytocin (τ = 0.295, p = 0.002). These associations, however, did not reach significance in FDR-adjusted analysis (p > 0.05).

## Discussion

The study showed novel interaction of opioids, T2D and metformin with specific microbiota in African American men with high burden of chronic disease. Previous studies implicated bifidobacteria and lactobacilli as playing important role in psycho-metabolic health [9–11]. This role was suggested to be explained at least in part by production of GABA, a well-known neuromodulator [12]. Therefore, we hypothesized that these bacteria could vary among the subgroups in our population with high burden of chronic psycho-metabolic disorders. The study supported at least in part this hypothesis. Connection to GABA production was further suggested by the interactive influence of opioids and metformin on microbiota in subjects with diabetes. The interactions of opioids and metformin were observed for genera *Bifidobacterium* and *Prevotella*, as well as unidentified species of *Prevotella* and *Bacteroides* caccae, all taxa previously reported as GABA producers [12].

### Interactions of opioids, T2D and metformin with Bifidobacterium

The study agreed with previous data suggesting associations of microbiota with T2D and metformin use although there were some discordant results [1,7,27]. More precisely, the current study suggested an association of T2D and metformin use with *Bifidobacterium*. *Bifidobacterium* abundance was significantly lower in subjects with T2D compared to those without T2D. This gram-positive anaerobe was previously shown depleted in patients with T2D [28] and T1D [29]. In T2D, an inverse association was observed between *Bifidobacterium* and high carbohydrate intake [30] and HbA1c [31]. Corresponding to depletion of *Bifidobacterium* in T2D, treatment of T2D patients with *Bifidobacterium* improved glycemic control suggesting probiotic property of this bacterium [11,32,33]. The current study also showed *Bifidobacterium* interacting with metformin in T2D, metformin treatment compared to no treatment was associated with higher abundance of *Bifidobacterium*. These data were in agreement with strong metformin signature in the human gut microbiome of T2D [27,34] and more specifically, with higher relative abundance of *Bifidobacterium* in T2D patients treated with metformin [34].

The current study showed significant difference in *Bifidobacterium* abundance when analysis was stratified by opioid use, T2D and metformin use, suggesting an interaction of these factors, i.e. the difference could not be explained by one factor alone and the effect of one factor was changed by the other. Previous studies demonstrated that the abundance of *Bifidobacterium* was lower in T2D individuals compared to controls. However, none showed that in patients with T2D using opioids, this effect was not observed. The T2D-opioid interaction could explain, at least in part, previously reported discordant results for associations of microbiota and T2D [1,7,27]. Considering wide-spread use of opioids in the general population and in T2D, this observation, if confirmed in larger studies, may be important as beneficial bacteria (probiotics) are suggested for improving diabetes care [3].

### Possible mechanisms of opioid interactions with microbiota

To date, no human cohorts have examined associations of opioid use with the gut microbiota. However, common gastro-intestinal side effects of opioids including nausea and constipation could possibly be attributed to changes in gut microbiota [20]. In mice, chronic morphine treatment significantly altered the gut microbial composition and induced preferential expansion of gram-positive Firmicutes and reduction in Bacteroidetes [19]. In various disease models describing morphine-mediated co-morbidities, morphine treatment caused changes in gut microbiota composition, dysregulated bile acids, disrupted intestinal mucosal immunity and integrity, and increased low grade and severe inflammation [19,20]. Moreover, transplantation of morphine-induced dysbiotic microbiome into healthy wild-type mice resulted in “morphine-like” diseased phenotype; whereas, transplantation of “normal” microbiome into morphine treated animals showed distinct improvement in the gut pathology suggesting causal relationship between morphine and microbiota changes [19]. Previously reported relationship between opioids and microbiota could also help explaining differences in bifidobacteria and lactobacilli abundance observed in the current study.

Previously, bifidobacteria as well as lactobacilli were identified as the most efficient producers of GABA [12], a neurotransmitter affecting pathophysiology of both T2D and psychiatric disorders [35, 36]. GABA-producing lactobacilli isolated from fermented dairy products (i. e. cheese, yogurt) were implicated in bioactive properties assigned to those foods [12]. The present study showed relatively low representation of lactobacilli, where this result could be explained by the previous observation in mice for morphine-mediated lowering of relative abundance of lactobacilli [19].

In addition to producing GABA, both bifidobacteria and lactobacilli can ferment complex carbohydrates into short chain fatty acids (SCFA, acetate, propionate, butyrate), known to beneficially impact metabolism (inducing intestinal glucagon-like peptide-1 [GLP-1] and PYY release) and behavior [3,15,27]. Specifically, in T2D patients, protein intake negatively correlated with *Bifidobacterium* abundance and SCFA production [30] while metformin use was associated with higher relative abundance of *Bifidobacterium* [34]. Consumption of probiotic fermented milk containing bifidobacteria and lactobacilli was shown to result in improved glycemic control compared to placebo in a double-blind, randomized, placebo-controlled trial of patients with T2D [11,32,33]. Moreover, supplementation of *Lactobacillus casei* resulted in enriched *Bifidobacterium* abundance, improved glycemic control and increased GLP-1 levels in mouse model of T2D [37]. However, at this time, there are no studies dedicated to mechanistic insight connecting opioid use with SCFA-producing microbiota.

Cross-sectional nature of the study precluded evaluation of causality and there have not been trials for *Bifidobacterium* use for opioid or other addiction disorders. In randomized controlled trials (RCTs) of healthy volunteers, however, combination of various bifidobacteria and lactobacilli strains (L+B) improved mood and anxiety [38], and reduced aggressive thoughts [38], but some results were discordant [6]. In another report, the (L+B) treatment in RCTs for T2D improved fasting blood glucose [32,33], increased insulin sensitivity [33], modestly decreased HbA1c [11,32], and reduced inflammation and oxidative stress [32]. These data suggested a potential link involving the gut microbiota, psychological factors, and metabolism that warrants consideration therapeutically.

### Possible mechanisms of opioid interactions with Bifidobacterium

The strongest association in the current study was observed in relation of opioids with the genus *Bifidobacterium*. Previous studies in mice showed that antibiotic-induced dysbiosis was associated with reduced Bifidobacterium spp. and downregulation of mu-opioid receptors in the gut [39] suggesting a possible connection of Bifidobacterium spp. with opioids. Of note, *Bifidobacterium* was among species strongly expressing activity of β-glucuronidase (GUS) enzymes [20]. Microbiome-encoded β-glucuronidase (GUS) enzymes had been found ubiquitously present in all major human microbiota phyla and play essential role in metabolizing xenobiotics [40]. Opioids similar to many other drugs were shown to undergo major biotransformation involving glucuronidation in the liver and subsequent hydrolysis by β-glucuronidase in both intestinal mucosal cells and gut bacteria [20]. Microbial β-glucuronidase was implicated in intestinal damage caused by nonsteroidal anti-inflammatory drugs (NSAIDs) and the widely used anticancer drug irinotecan that was blocked by GUS-targeted inhibitors [41,42]. Similar mechanisms could be proposed for explaining morphine-microbiota interactions. Diet also influenced β-glucuronidase activity [20,43]. For example, fecal microbial β-glucuronidase activity was increased in people consuming high-meat diet compared with diets without meat [43]. Contrary, high-vegetable diet enhanced biomass of bifidobacteria and was associated with reduced microbial β-glucuronidase activity [44]. Diet was not evaluated in the present study, however, based on previous data in comparable population [7] we did not expect major influence of diet, as each group likely had very similar dietary patterns. Mechanistic role of gut microbiota in opioid metabolism and its role in opioid sensitivity and addiction remains an unknown question worthy of greater investigation.

### Possible mechanisms of opioid-metfromin interaction with Bifidobacterium

*Bifidobacterium* genus was also significantly affected by opioids and metformin interaction. The abundance of *Bifidobacterium* was significantly different in subjects using opioids compared to those not using opioids if they were not taking metformin, yet this effect was not observed in the presence of metformin. Conversely, metformin action on *Bifidobacterium* differed by the presence or absence of opioids. In subjects using opioids, metformin was associated with decreased *Bifidobacterium*. Contrary, in subjects not using opioids, metformin was associated with increased *Bifidobacterium*. While no previous studies have shown these interactions, a study evaluating metformin tolerance in more than 400,000 patients showed that among 28 drugs expected to interfere with metformin metabolism, codeine was the only drug significantly associated with early discontinuation likely due to metformin intolerance [45]. Codeine, similar to other opioids, is an inhibitor of organic cation transporter 1 (OCT1) [45]. OCT1, acting in the liver and intestinal cells, is important for metformin pharmacokinetics and therapeutic efficacy, and could be involved in 80-fold variability of steady-state metformin concentration reported in T2D patients [46]. Of note, we observed lower HbA1c in the subgroup with T2D on MF and taking opioids vs. those not taking opioids. This observation could possibly be explained by opioids acting as OCT1 inhibitors and leading to higher blood and/or tissue level and efficacy of metformin. OCT1 inhibitors were suggested as contributors to gastrointestinal side effects and intolerance of metformin experienced by up to 20–30% of patients [47,48]. The data from the current and previous studies suggest the possible importance of OCT1 for metformin and opioid metabolism, implying that OCT1 could be a mechanistic link for interaction of metformin and opioids observed in the current study.

### Leptin and oxytocin associations with Dialister

In addition to *Bifidobacterium*, *Dialister* featured prominently in relationship with selected key biomarkers. There was a trend for association of *Dialister* with circulating leptin and oxytocin in subjects without diabetes. Specifically, lower abundance of *Dialister* was seen in High-Leptin compared to Low-Leptin subgroup, while the opposite direction of relationship was seen for oxytocin. Leptin and oxytocin have each been strongly associated with obesity and T2D [16,17] and psychiatric conditions [49,50] in previous studies. The possible differing relationship of *Dialister* with leptin and oxytocin could be explained at least in part by opposite association of these hormones with obesity and T2D reported in previous studies [16,17]. In the present study, leptin was increased in subjects with T2D compared to those without T2D while oxytocin was not different among the subgroups. Corresponding with reported data [1], we previously observed higher abundance of *Dialister* associated with higher glucose level in prediabetes [7]. Similarly, *Dialister* abundance positively correlated with dietary carbohydrates [51], supporting important role of *Dialister* in glucose metabolism. In addition, association between oxytocin (as a behavioral hormone) and *Dialister* shown in this study agreed with previously reported association of *Dialister* with behavioral characteristics in young children [5], implicating *Dialister* in possible impact on brain function. Taken together, the data from us and others supported a mechanistic role of the gut microbiota in the gut-brain axis effects on physical and mental health.

### Limitations and conclusions

The study has limitations. The 16S rRNA sequencing for taxonomic profiling has relatively limited resolution and a narrower range than metagenomic approaches [52]. The correlational nature of the analyses does not allow determining if observed interactions and associations are a function of effects of opioid on the gut microbiota, effects of microbiota on opioid metabolism, or a combination thereof. Diet is known to be a major contributor to microbiota composition and was not evaluated here. Lifestyle behavior including smoking and physical activity can be additional potential confounders. Similarly, specificity of the population, e.g. high burden of disease prevents generalizing the findings to other populations.

In conclusion, the present study showed possible physiological links between microbiota and brain in agreement with previous human and animal data. The data showed novel interactions of microbiota with opioids, T2D, and metformin, suggesting possible venues for the management of T2D with psychiatric co-morbidities by targeting the gut microbiota. The data also corroborated previous research implying that some specific probiotic bacteria could be of importance to the host health. The report contributed to a growing literature linking gut microbiota to human behavior and metabolism. Further studies including randomized trials are needed to provide relevant clinical outcomes and mechanistic insights into gut microbiota-brain connections.

## Supporting information

S1 File The primary data set. The bio-clinical subjects characteristics.
(XLSX)
